# Prevalence and Associated Factors for Dual Form of Malnutrition in Mother-Child Pairs at the Same Household in the Gaza Strip-Palestine

**DOI:** 10.1371/journal.pone.0151494

**Published:** 2016-03-18

**Authors:** Rima Rafiq El Kishawi, Kah Leng Soo, Yehia Awad Abed, Wan Abdul Manan Wan Muda

**Affiliations:** 1 Program of Nutrition, School of Health Sciences, Health Campus, Universiti Sains Malaysia, Kubang Kerian, 16150 Kelantan, Malaysia; 2 School of Public Health, Al Quds University, Gaza City, Gaza Strip, Palestine; University of Ottawa, CANADA

## Abstract

**Background:**

In many developing countries nutritional, and epidemiological transitions are emerging into continuing undernutrition and escalating overnutrition, giving rise to the double burden of the malnutrition phenomenon.

**Objectives:**

This study aims to determine the prevalence of the dual form of malnutrition (overweight mother/underweight child) in the same household and its associated factors in the Gaza Strip.

**Methods:**

A total of 357 mother-child pairs from the same households were surveyed from three different geographical locations in the Gaza Strip, namely, El Remal urban area, Jabalia refugee camp, and Al Qarara rural area. The height and weight of mothers aged 18–50 years were measured, and their body mass index (BMI) was computed. The mothers were categorized according to the criterion of the World Health Organization (WHO) for BMI as overweight if they have a BMI ≥ 25 kg/m^2^. Anthropometric indices were measured for children aged two to five years to classify the underweight children Z-score <-1.

**Results:**

The results showed the prevalence of the dual form of malnutrition in the Gaza Strip was 15.7%, and its associated factors were child’s birth order (OR_adj_, 1.50, 95% CL, 1.22, 1.82; *p* = <0.001), father’s educational (low or medium) levels (OR_adj_, 3.19, 95% CL, 1.07, 9.5; *p* = 0. 036), or (OR_adj_, 3.4, 95% CL, 1.12, 10.37; *p* = 0. 031), high scores of mothers' nutrition knowledge (OR_adj_, 1.23, 95% CL, 1.01, 1.52; *p* = 0. 048), and low monthly income (OR_adj_, 0.28, 95% CL, 0.09, 0.88; *p* = 0. 030).

**Conclusions:**

The results from this study showed the dual form of malnutrition in the same household was prevalent in the Gaza Strip. This is a public health issue that must be understood and addressed and policy makers must implement an appropriate nutrition action plan to control dual form of malnutrition based on the underlying specific risk factors in the study population. In addition, interventions are needed to help individuals to translate their nutrition knowledge into healthy dietary behaviors.

## Introduction

In developing countries nutrition transition is a continuous process that occurs in all societies as patterns of food consumption and physical activity change over time [1]. In low and middle income countries, urbanization and economic development have propelled a change towards “westernized” diet and lifestyle. Nutrition transition is characterized by a transition from traditional diets, which mostly derive from plant-based food sources requiring high labour intensity, toward more varied diets containing more refined, energy dense, animal based and processed food which are generally high in sugar and fat and low in carbohydrate content [2]. These dietary changes are also accompanied by decreasing physical activity in the form of walking or cycling and increasing sedentary time in offices [3]. Nutrition transition has a positive effect on reducing infectious diseases and child mortality. It has also led to an increase in the proportion of overweight and obese individuals as well as non-communicable diseases (NCDs) related to nutrition [4]. Middle Eastern countries have been undergoing a fast paced nutrition transition [5]. The high obesity rates are prevalent among adults accompanied by undernutrition among children and these two issues may even coexist at the household level to produce the so-called dual form of malnutrition [6,7]. Recent evidence indicates that an underweight child and an overweight mother may coexist in close proximity. These conditions may occur at national, community and even household levels [8]. The most common combination of dual burden households observed was paradoxically underweight children and overweight mothers [9]. Underweight reflects both in wasting and stunting. Moreover, underweight is the most common assessment method used for monitoring the child’s growth to assess changes in the magnitude of malnutrition over time [10]. Some studies have used stunting instead of underweight as the nutritional indicator for malnourished children [7]. National surveys from Brazil, China, and Russia revealed 11.0% dual form of malnutrition prevalence among Brazilian households and 8.0% dual form of malnutrition prevalence among Chinese and Russian households [8]. The Palestinian community, like other countries, is experiencing rapid changes in diet and physical activity. Parallel to malnourished children, studies have shown that obesity and nutrition-related chronic diseases are prevalent among Palestinian adults [11].

Findings of increasing prevalence of obesity and overweight among adults and underweight among preschool children lead to the hypothesis that overweight and underweight might cluster within the same household. Previous studies supported the presence of the dual form of malnutrition in the Palestinian community [12]. The dual form of malnutrition in the same household is an important concern for public health intervention. Undernutrition intervention aimed at reducing an underweight person is likely to increase food availability to the whole household. Thus, there may be an increased risk of overweight and obesity to other individuals in the household. Contrariwise, overweight interventions may reduce fat diet, which may have negative impacts on any underweight members in the same household [6,8].

The aim of this study was to determine the dual form of malnutrition and its associated factors in the Gaza Strip.

## Materials and Methods

### Study design

This cross-sectional study was conducted in three different areas in the Gaza Strip, namely, El Remal urban area, Jabalia refugee cam and Al Qarara rural area. It was carried out from April to October 2012.

### Sample size

The sample size was calculated using the single proportion formula [13], as follows:

The anticipated population proportion (*P*) was 32.0%, which was for obesity prevalence among women in the Gaza Strip [14]. The level of significance was 95.0%.

$$n\;=\;Z^{2}\;P\;\left(1\;-\;P\right)/d^{2}$$

Where n is the sample size

*Z* is the *Z* statistic for a level of confidence of 1.96

P is the expected prevalence or proportion of 32.0%

d is the precision (d = 0.05)

$$\text{n }=\;{\left(1.96\right)}^{2}\;\times \;0.32\;\left(1\;-\;0.32\right)\;/\;{\left(0.05\right)}^{2}\;=\;334.$$

Accounting for an attrition rate of 20.0%, the total number of participants was calculated as follows: 334 + (0.20 × 334) = 400. Finally, 400 participants were recruited for the study.

### Sampling method

A total of 400 households were screened to find individuals who met the inclusion and exclusion criteria which were: a mother aged 18 to 50 years who was not pregnant, and a child aged 2 to 5 years who did not suffer from psychomotor retardation, hormonal disorders, chronic debilitating diseases, congenital heart diseases, or acute severe illnesses. Depending on the sociodemograhic situation, three clusters were selected, namely, Jabalia refugee camp in the north of the Gaza Strip, El Remal in Gaza city, and Al Qarara in the south of the Gaza Strip. At the first stage, numbers of areas were selected randomly from the entire urban area, refugee camp, and from the rural cluster. At the second stage, households were systematically selected within each cluster with proportional to the total population of women aged 18–50 years (19.1%). A total of 220, 140 and 40 households were selected from Jabalia refugee camp, El Remal, and Al Qarara, respectively; mothers and children were then recruited from the selected households. In households with more than one child aged 2 to 5 years, the youngest child was selected for the child-mother pairs.

### Data collection

A structured questionnaire was used to collect information on several sociodemographic and economic profiles at the individual and household levels, and nutrition practice. The structured questionnaire was validated by taking expert opinions from seven professionals in the field of health and nutrition, and the necessary revisions were made based on their advices and comments. Prior to conducting the study, the questionnaire was piloted on 30 women and after obtaining the responses, the necessary changes were made and the responses were evaluated. Moreover, the researcher used questions that had been predesigned (mother’s nutrition knowledge). The nutrition knowledge was evaluated using a 16-item questionnaire adapted from the National Coordinating Committee for Food and Nutrition, Ministry of Health, Malaysia, 1997. The questionnaire related to sources of nutrients, types of healthy food, healthy food and body functions, obesity and exercise, and food and diseases. The questionnaire had to be forwardly and backwardly translated into Arabic and then English. The questionnaire was circulated among experts in health and nutrition from Al-Quds University, and the United Nations Children's Fund (UNICEF) in the Gaza Strip to test its understandability, acceptability, and appropriateness. All comments were revised and some minor corrections made as needed. Kuder-Richardson Formula 20 (KR-20) was used to measure internal consistency of measurements for dichotomous choices. The result of KR-20 coefficient was 0.76, which was considered acceptable, as the acceptable values can range from 0.00 to 1.00. The median was used as the numeric value separating the higher half of a mothers’ scores distribution, from the lower half (the median = 13).

To assess nutrient intake for the child-mother pairs, the researcher used 24-hour recall. The researcher asked the mothers to recall all of the food and beverages consumed over two days, that is, one day before the interview and the second one was the weekend for variation in food intake. Nutritionist Pro software, version 1.2, was used. The nutrition composition tables were selected based on their suitability for the actual composition of food available in the area where the data were collected. All food items consumed are expressed in grams or milliliters, based on the Recommended Dietary Allowance (RDA) [15].

The heights and weights of mothers and children were measured using the standard recommended procedures of the World Health Organization (WHO). A SECA digital weighing scale (to the nearest 0.1 kg) was used to measure weights. The child was weighed barefoot, wearing only underwear. While, the mother was weighed with light clothes on, and without shoes. The researcher calibrated the scale before each measurement session. Accuracy was checked by comparing the scale reading with a known weight. The measurements were taken twice and the average was calculated. Heights were measured using a plastic, non-stretchable constant tapeline, with 0.1 cm precision, affixed to a plain vertical surface. The child was instructed to remove his/her shoes. The height was then measured while standing against a wall with feet flat on their base, the heels, buttocks, shoulders, and back of the head touching the wall, and the head positioned looking straight ahead. The mother stood without shoes against a wall, with feet flat on their base, the heels, buttocks, shoulders, and back of the head touching the wall, and the head positioned looking straight ahead. The mean of two measurements was calculated. The Body Mass Index (BMI) was computed for each mother with the following formula: weight (kg)/height (m)^2^. According to the WHO, an adult BMI of <18.5 is underweight, 18.5–24.9 is normal weight, and ≥ 25.0 is overweight [16]. The ages of the children were calculated in months from their birth certificates. Raw anthropometric data for children were transformed into Z-scores using the program WHO ANTHRO (2.2, January 2011). To assess children’s nutritional statuses, Z-scores were calculated according to the WHO child growth standards for children. Underweight child was defined as weight -for- age Z scores with WAZ of children were classified as underweight <-2 SD [17], and underweight <-1 SD [18].

### Ethical issues

Ethical approval was obtained from the Helsinki Committee in the Gaza Strip, and ethical approval was approved from Universiti Sains Malaysia (USM) ethical committee. The written consent form was obtained from each mother before the interview. Written permissions of the mothers were required for anthropometric measurements for their children. Moreover, mothers were informed that the questionnaires could be answered voluntarily and anonymously and that the information would be treated confidentially.

### Data analysis

The edited and completed data set was entered using SPSS, version 20.0. We used frequency and proportion to describe the characteristics of the study sample and to estimate the prevalence of the dual form of malnutrition in the Gaza Strip. Binary logistic regression was performed to control for confounding factors. In this model the dependent variable was household with the dual form of malnutrition (overweight mother/underweight child).

While the independent variables were household sociodemographic characteristics as follow:

1. Geographical location (urban, rural, or refugee camp).
2. The size of the household (number of family members).
3. Father’s educational level: We coded this variable into elementary or below and preparatory (low level), secondary (medium level), graduate (diploma/university level), and postgraduate (master’s degree/PhD) (high level), the same classification used for mothers’ education level.
4. Food or money assistance was received from nongovernment agencies like UNRWA (United Nations Relief and Works Agency).
5. Family monthly income was expressed in New Israel Shekel (NIS). NIS 3.9 was equal to US$1.

Child’s characteristics

The child’s mother was interviewed for information regarding the child. These include:

1. Age in months obtained from the birth certificate.
2. Child’s sex.
3. Birth order with regard to his/her siblings.
4. Nutrition practice: Exclusive breast feeding (breastfeeding up to six months) [19], the appetite of the child, number of meals.

Mother’s characteristics

1. Age
2. Number of pregnancies
3. Nutrition knowledge
4. Nutrients intake for mother-child pair

In the simple logistic regression (SLgR), we examined the relationship between each of the independent variables and the dependent variable. The independent variables with *p* values <0.25 and logically related to the dual form of malnutrition that were tested in SLgR, and then were included in the binary logistic regression (MLgR). A logistic model was subsequently considered using a backward test for the final model of the dual form of malnutrition with a significance level of *p* < 0.05.

## Results

Among the 400 households, 357 were recruited (response rate: 89.2%). Of the 43 non-respondents, 12 mothers refused to participate in the study, 9 mothers were pregnant, 8 households were excluded as the children were aged under two years, and 14 children refused anthropometric measurements. Table 1 presents the different forms of nutritional status combinations of child-mother pairs: Mean weight of all children was 14.20 (2.42) kg, and mean height was 94.14 (7.94) cm. The mean weight of all mothers was 70.95 (15.46) kg, while the mean height was 1.59 (0.05) m, with a mean BMI of 27.90 (5.91) kg/m^2^. About 64.1% of the mothers were overweight, while 33.9% of the mothers had normal body weight. The mean body weight of the overweight mothers in the OWM/UWC group was higher at 74.93 (13.12) kg than that of the NWM/NWC group at 58.42 (5.60) kg. About 24.4% of the children were underweight (Z-score<-1.0), these percentages included both mild and moderate malnourished. The mean body weight of the underweight children in the OWM/UWC group was lower at 12.25 (1.64) kg than the normal body weight of the children in the NWM/NWC group at 14.20 (1.87) kg. Table 2 shows the classification of households with dual form of malnutrition overweight mother/underweight child, according to the nutritional status of children. Reuslts revealed that the prevalence of dual form of malnutrition was 2.5% when overweight mother (BMI≥ 25.0) / underweight child (<-2 SD) [17], while the prevalence of household with dual form of malnutrition was 15.7% when overweight mother (BMI≥ 25.0) / underweight child (<-1 SD) [18].

**Table 1:** Anthropometric characteristics of child—mother pairs.

	Total (n = 357)		OWM/UWC (N = 56)		NWM/NWC (n = 90)	
	n (%)	Mean(SD)	n (%)	Mean (SD)	n (%)	Mean (SD)
**Mothers**						
Weight (kg)		70.95 (15.46)		74.93(13.12)		58.42(5.60)
Height (m)		1.59 (0.05)		1.56(0.06)		1.60(0.05)
BMI (kg/m^2^)		27.9 (5.91)		30.31(4.63)		22.60(1.68)
Underweight (BMI<18.5)	7 (2.0)		0 (0.0)		0 (0.0)	
Normal weight (18.5–24.99)	121 (33.9)		0 (0.0)		90 (100.0)	
Overweight (BMI≥ 25.00)	229 (64.1)		56 (100.0)		0 (0.0)	
**Children**						
Weight(kg)		14.20(2.42)		12.25(1.64)		14.20(1.87)
Height(cm)		94.14(7.94)		91.07(7.04)		93.51(6.80)
Weight-Age (Z-score)		-0.48 (0.99)		-1.75(0.42)		-0.18 (0.67)
Significant underweight (<-2.0)	13 (3.6)					
Mild underweight (-2.0-to<-1.0)	74 (20.8)					
Underweight < -1	87 (24.4)		56 (100.0)		0 (0.0)	
Norma weight (-1.0-to 2.0)	267 (74.8)		0 (0.0)		90 (100.0)	
Overweight>2	3 (0.8)		0 (0.0)		0 (0.0)	

OWM/UWC: Overweight mother (BMI≥ 25. 0) /underweight child (< -1SD) indicated paradoxical burden malnutrition group

NWM/NWC: Normal weight mother/normal weight child indicated normal group

**Table 2:** Classification of households with dual form of malnutrition overweight mother/underweight child according to nutritional status of child.

Characteristics	Frequency (n)	Percent (%)
Overweight mother (BMI≥ 25.0) /Underweight child (Z-score<-2) * (OWM/UWC)	9	2.5
Overweight mother (BMI≥ 25.0) /Underweight child (Z-score -2.0-to<-1.0) (OWM/UWC)	47	13.2
Overweight mother (BMI≥ 25.0) /Underweight child (Z-score <-1) ** (OWM/UWC)	56	15.7

*WHO,2006

**WHO, 1983

Table 3 presents the frequencies and percentages of households, according to nutritional status of mother-child pairs. The highest prevalence (48.1%) was overweight mother/normal weight child. The prevalence of overweight mother/underweight child pairs [OWM(BMI≥ 25.0) **/** /UWC(<-1 SD)] was 15.7%, the prevalence of normal weight mother/normal weight child (NWM/NWC) was 25.2%, while the prevalence of normal weight mother/underweight child (<-1 SD) was 8.1%. In Tables 4 and 5 we compared the general characteristics of the participants in households with the dual form of malnutrition [overweight mother (BMI≥ 25.0) **/** underweight child (<-1 SD)] to the normal weight mothers/normal weight children. The majority of the participants in the two groups OWM/UWC and NWM/NWC were more likely to be found in the Jabalia refugee camp (64.3% and 60.0% respectively). No significant difference was observed in the geographical locations between the two groups. Our results showed a high proportion of participants lived in poor households and no significant difference was found. This piece of data indicated that most mothers and children were from a deprived socioeconomic background. Similarly, no significant differences were observed between OWM/UWC and NWM/NWC pairs in terms of household characteristics such as: father’s job, mother’s job, and household assistance. But results revealed that comparing the mean difference (MD) of mothers’ age between OWM/UWC 31.55 (6.62) year and NWM/NWC 27.93 (5.27) year was statistically significant (*p*<0.001). Additionally, the mean difference (MD) of child’s birth order between OWM/UWC 4.43 (2.35) and NWM/NWC 2.94 (1.88) was statistically significant (*p* = 0. 028). The mean difference (MD) of the number of children in the household between OWM/UWC 6.96 (1.90) and NWM/NWC 5.60 (1.67) was statistically significant (*p* <0.001). Moreover, an association was found between the mother’s educational level in the two groups OWM/UWC and NWM/NWC (x^2^ = 6.81; *p* = 0.033). Results revealed that there was an association between child’s appetite and groups of OWM/UWC and NWM/NWC (x^2^ = 9.13; *p* = 0.010). Table 6 shows the mean difference (MD) of mothers’ fat intake between overweight mothers in the households with dual form of malnutrition 70.73 (51.10) and normal weight mothers 59.04 (37.17) was statistically significant (*p* = 0.044). Results revealed that when comparing the energy and macronutrients intake (protein, and carbohydrate) between the two categories of overweight mothers lived in the households with dual form of malnutrition, and normal weight mothers, the difference between proportions wasn’t statistically significant, but overweight mothers lived in the households with dual form of malnutrition consumed higher energy and macronutrients than the others. The underweight children lived in the households with dual form of malnutrition consumed lower energy and macronutrients than normal weight children, and there was no statistically difference. A binary regression analysis was done with the outcome and explanatory variables described in Table 7. Univariate analysis was done first with all explanatory variables of interest followed by binary regression analysis. A binary logistic regression model was developed to determine the associated factors of the dual form of malnutrition 56 overweight mothers/ underweight children [OWM (BMI≥ 25.0) /UWC (<-1 SD)] with 90 normal weight mother/normal weight children (NWM/NWC). Table 7 presents the results of simple logistic regression (SLgR). The reference group for all analyses is normal weight children/normal weight mother pairs, which allows us to predict the influence of numerous risk factors on the prevalence of paradoxical pairs as compared to the normal group. Several factors can be thought of as likely determinants of intra-household nutritional inequality. These include the sociodemographic, mother’s variables, child’s variables, and nutrient intake. Based on SLgR, independent variables with p-value <0.25 or any other important associated factors from previous studies were included in binary Logistic Regression MLgR. The preliminary final effect model was obtained using backward LR variable selection. Results in Table 7 showed the associated determinants appearing in the final model that remained significantly associated with the dual form of malnutrition, namely, child’s birth order, father’s education, family income, and mother’s nutrition knowledge. Child’s birth order was significantly associated with the dual form of malnutrition (OR_adj_, 1.50, 95% CL, 1.22, 1.82; *p* = <0.001). Households with father’s education level low or medium were more likely to have the dual form of malnutrition (OR_adj_, 3.19, 95% CL, 1.07, 9.50; *p* = 0. 036), or (OR_adj_, 3.40, 95% CL, 1.12, 10.37; *p* = 0. 031), respectively, than households with father with high education level. Poor households were less likely to have the dual form of malnutrition (OR_adj_, 0.28, 95% CL (0.09, 0.88); *p* = 0.030). The finding of the study revealed that the dual form of malnutrition increased with increasing scores of woman nutrition knowledge (OR_adj_, 1.23, 95% CL, 1.01, 1.52; *p* = <0.048).

**Table 3:** Classification of households according to nutritional status of mother/child pairs.

Characteristics	Frequency (n)	Percent (%)
Overweight mother/Underweight child (OWM/UWC)	56	15.7
Normal weight mother/Normal weight child (NWM/NWC)	90	25.2
Overweight mother/Normal weight child (OWM/NWC)	172	48.1
Normal weight mother/Underweight child (NWM/UWC)	29	8.1
Underweight mother/Underweight child (UWM/UWC)	2	0.6
Underweight mother/Normal weight child (UWM/UWC)	5	1.4
Overweight mother/ Overweight child (OWM/OWC)	2	0.6
Normal weight mother/Overweight child (NWM/OWC)	1	0.3

**Table 4:** Sociodemographic characteristics of normal weight mother/normal weight child and overweight mother/underweight child pairs.

	NWM/NWC (n = 90)		OWM/UWC (n = 56)				
Variables	n (%)	Mean(SD)	n (%)	Mean(SD)	*t-stat*(df) *x*^2^ (df)	Mean Difference (95% C.I.)	P-value
**Geographical location**							
City	28(31.1)		17(30.4)				0.712^†^
Rural	8(8.9)		3(5.3)				
Refugee camp	54(60.0)		36(64.3)				
**Father's educational level**							
Low	31(34.4)		23(41.1)				0.191^†^
Medium	20(22.3)		17(30.3)				
High	39(43.3)		16(28.6)				
**Father's job**							
Working	64(71.1)		46(82.1)				0.168^†^
Not working	26(28.9)		10(17.9)				
**Household’s member**		5.6(1.67)		6.96(1.90)	-4.53 (144)	-1.36 (-1.96,-0.77)	<0.001^††^
Family income (Shekel)*							
>2000	15(16.7)		15(26.8)				0.232^†^
1000–2000	32(35.5)		21(37.5)				
<1000	43(47.8)		20(35.7)				
**Household with assistance**							
Yes	46(51.1)		25(44.6)				0.498^†^
No	44(48.9)		31(55.4)				

^††^ Independent t test.

^†^ Pearson Chi-Square. Significant level p-value< 0.05

NWM/NWC:Normal weight mother/Normal weight child. OWM/UWC:Overweight mother/Underweight child

*1USD$ = 3.9Shekel

**Table 5:** Mother &chid characteristics of normal weight mother/normal weight child and overweight mother/underweight child pairs.

	NWM/NWC (n = 90)		OWM/UWC (n = 56)				
Variables	n (%)	Mean(SD)	n (%)	Mean(SD)	*t-stat*(df) *x*^2^ (df)	Mean Difference (95% C.I.)	P-value
**Mother’s age (year)**		27.93(5.27)		31.55(6.62)	-3.65 (144)	-3.62(-5.58,-1.66)	<0.001^††^
**Mother’s educational level**							
High	27(30.0)		13(23.2)				0.033^†^
Medium	40(44.4)		17(30.4)				
Low	23(25.6)		26(46.4)				
**Mother’s Job**							
Employment	4(4.4)		6(7.1)				0.483^†^
Household	86(95.6)		52(92.9)				
**Mother’s nutrition knowledge**		12.26(2.29)		12.70(1.80)	-1.19 (144)	-0.49(-1.14,0.28)	0.236^††^
**Child’s age (month)**		37.05(10.13)		40.51(10.56)	-1.97 (144)	-3.64(-6.92,0.04)	0.050^††^
**Child’s sex**							
Male	44(48.9)		30(53.6)				0.613^†^
Female	46(51.1)		26(64.4)				
**Child’s birth order**		2.94 (1.88)		4.43 (2.35)	-4.20 (144)	-1.48(-2.18,-0.78)	<0.001^††^
**Child’s appetite**							
Good	35(38.9)		12(22.4)				0.010^†^
Fair	35(38.9)		19(33.9)				
Poor	20(22.2)		25(44.7)				
**Exclusive Breastfeeding**							
Yes	24(26.7)		11(19.6)				0.426^†^
No	66(73.3)		45(80.4)				

^††^ Independent t test.

^†^ Pearson Chi-Square. Significant level p-value< 0.05

NWM/NWC:Normal weight mother/Normal weight child. OWM/UWC:Overweight mother/Underweight child

**Table 6:** Nutrients intake among mother-child pairs in households with dual form of malnutrition and normal households.

Nutrients	Households with dual form of malnutrition (n = 56)	Normal Households (n = 90)	Mean diff. (95% C.I.)	t-statistic (df)	p-value
	Mean (SD)	Mean (SD)			
Mothers					
Energy (Kcal)	1448.67 (348.78)	1430.00 (281.05)	-18.43(-102.17, 65.30)	-0.43 (144)	0.665
Protein (g/d)	52.01 (20.66)	50.80 (20.58)	-1.20 (-7.10, 4.68)	-0.40 (144)	0.687
Fat (g/d)	70.73 (51.10)	59.04 (37.17)	-11.68 (-25.97, 2.60)	2.11 (144)	0.044
Carbohydrate (g/d)	152.53 (78.48)	148.73 (58.20)	-3.80 (-21.48, 13.88)	-0.42 (144)	0.673
Children					
Energy (Kcal)	897.00 (322.78)	910.56 (385.14)	12.70 (-94.95, 120.36)	0.23 (144)	0.817
Protein (g/d)	31.31 (17.25)	35.25 (21.93)	3.94 (-2.14, 10.03)	1.27 (144)	0.203
Fat (g/d)	33.25 (15.40)	34.11 (16.94)	0.89 (-3.90, 5.67)	0.36 (144)	0.715
Carbohydrate (g/d)	110.60 (64.05)	112.47 (76.54)	0.86 (-20.53, 22.25)	0.08 (144)	0.937

Independent t test. Significant level p-value< 0.05

**Table 7:** Associated factors of dual form of malnutrition in the same household.

Variables	Simple Logistic Regression			Multiple Logistic Regression		
	B	Crude OR (95%CI)	P-value	Exp(B)	Adjusted OR (95.0% C.I.)^b^	P-value
Child birth order	0.33	1.39 (1.17,1.65)	<0.001	0.40	1.50 (1.22,1.82)	<0.001
Father’s medium educational level	0.72	2.07 (0.86,4.94)	0.101	1.16	3.19 (1.07,9.50)	0.036
Father’s low educational level	0.59	1.80 (0.86,3.99)	0.143	1.22	3.4 (1.12,10.37)	0.031
Family low income (Shekel)	-0.76	4.65 (0.19,1.13)	0.092	-1.26	0.28 (0.09,0.88)	0.030
Mother’s nutrition knowledge	-0.10	1.10 (0.93,1.30)	0.236	0.210	1.23 (1.01,1.52)	0.048

^b^Backward LR Binary Logistic regression model was applied.

Educational High level is the reference Diploma, university level, and master’s degree/PhD

Educational Medium level: secondary

Educational Low level: elementary or below and preparatory

High Income is the reference > 2000NIS (1$US = 3.9Shekel)

## Discussion

This study presents evidence of a dual-pronged problem of malnutrition, which consists of underweight children and overweight/obese mothers living in the same household. The Palestinian community, like other countries, is experiencing rapid changes in diet and activity. The prevalence of hypertension, diabetes mellitus, and tobacco smoking are now higher and similar to those in neighbouring countries [20]. Often these changes occurred despite persistent undernutrition among children [21]. In the Gaza Strip, most of the households live in poverty because of the socioeconomic deprivation, which is a result of its isolation from the outside world [22]. This deprived community showed the coexistence of the dual form of malnutrition, namely, underweight children and overweight mothers. There were no previous studies conducted in Palestine on the pattern of the dual form of malnutrition or its determinants in the same households. Since the number of underweight children defined by WAZ <-2SD was small (only 13), thus for this study it was decided that, the underweight children would include all children whose WAZ < -1SD [3]. Moreover, the worsening conditions in the Gaza Strip have negatively affected the population, particularly children. Thus, due to the deteriorated situation in the Gaza Strip the researcher used Z-score to identify underweight (WAZ<-1SD). Our results revealed that 15.7% of households in the Gaza Strip coexisted with overweight/underweight mother-child pairs, while approximately half of the households coexisted with overweight mother/normal weight child. These findings may be attributed to the high prevalence of overweight mothers and normal weight children among participants. The paradoxical coexistence of adult overweight with childhood malnutrition has been documented in a number of communities [9]. Survey data showed the coexistence of overweight and underweight in the same household is most prevalent in Brazil 11.0%, China 8.0%, and Russia 6.0% [8]. Among the indigenous people in Malaysia, 25.8% of the surveyed households showed the co-existence of underweight children and overweight mothers in the same household [23]. A recent study revealed that 29.6% of households in a rural district of Malaysia can be classified as having an underweight child/overweight mother [24]. The increase in child birth order also increases the risk for underweight, whereas the adults tended to be overweight within the same household, which may suggest repeated pregnancies and possibly less care for individual children [25]. These findings are consistent with other studies that reported child birth order as an important factor influencing nutritional status of children. Perhaps mothers with many children have less time to extend equal care to each of the children compared with those who have fewer children [26,27]. Fathers’ educational background also emerged as an important factor associated with underweight status among children under five years old [28]. Findings revealed that fathers’ education levels are negatively associated with the dual form of malnutrition. Households with fathers of low and medium educational levels showed significantly higher manifestations of the dual form of malnutrition. Diets of preschool children aged two to five are largely dependent on foods supplied by parents and other adults [29]. Previous studies conducted in Bangladesh showed that children whose fathers had higher levels of education had a lower rate of weight deficiency than those with illiterate fathers. The father is usually the main earner and decision maker of a family, so a higher level of education may play an important role in ensuring better nutritional status for the children [28]. The results of this current study showed a significant association between the dual form of malnutrition and household income. Curiously, high-income households may be at higher risk of the dual form of malnutrition. A possible explanation is that households with higher income may enter the nutrition transition and adopt Western lifestyles more rapidly. Producing changes in dietary and activity patterns also increases the risk of overweight and obesity, whereas many of the risk factors for undernutrition still remain [7]. These findings are consistent with those of other studies, in which one aspect that has received particular attention is the relationship between the dual form of malnutrition and income. In Indonesia and China, for example, dual burden households generally have higher average incomes than the rest; in other countries, such as Brazil and Russia, dual burden households tend to have lower than average incomes [30]. The current study reported that the proportion of households with overweight mother/underweight child was positively associated with the mothers’ nutrition knowledge. In the final model, after adjusting for confounding factors such as the mother’s age, results revealed that good knowledge on the part of the mothers was associated with poor food practices for children and themselves. The mother’s nutrition knowledge did not translate into healthy practice; therefore, nutrition knowledge of mothers was associated with increasing malnutrition among individuals at household level. These findings are consistent with another study, which reported that good knowledge failed to convert into positive changes in food behavior [31].

### Study limitations

The study design was cross-sectional to identify associated factors with the dual form of malnutrition, which could not determine the cause and effect relationship. Case control studies should be conducted in the future to address the risk factors of the dual form of malnutrition in the Gaza Strip. For 24-hour recall analysis, the results were based on estimates; therefore, they may give rise to recall bias.

## Conclusion and Recommendation

The coexistence of maternal overweight with child undernutrition in the same household is a health concern in the Gaza Strip. The socioeconomic situation of the household and birth order of the child appears to be important predictors of the dual burden of malnutrition. That highlights the importance of having effective strategies within families, focusing on nutrition education and the effective guidance to help parents promote caring and proper eating patterns among their children. In addition, maternal nutrition knowledge is also an important indicator for underweight/overweight households. Thus, interventions are needed to help individuals to translate their nutrition knowledge into healthy dietary practices. This is the first study conducted in the Gaza Strip, thus the results shed light on the public health concern of the dual form of malnutrition in the same household in the Gaza Strip. The sample of this study was selected randomly from three different sociodemograhic areas in the Gaza Strip. Thus, the results of this study can be generalized to the whole population, however, we are aware our sample is small. Further studies with larger sample sizes are needed to develop programs that could effectively address the existence of the dual form of malnutrition on a population’s health and nutrition. It is important to investigate these determinants of the underweight-overweight pairs at the same household for planning successful nutrition intervention programs that consider the coexistence of two individuals representing the opposite types of malnutrition (underweight and overweight) while sharing the same household environment.
